# In the shadow of the healing rainbow: belonging and identity in the regulation of traditional medicine in Mauritius

**DOI:** 10.1080/10383441.2023.2249708

**Published:** 2023-08-26

**Authors:** Nayeli Urquiza-Haas, Emilie Cloatre

**Affiliations:** a Law, Lancaster University, Lancaster, UK; b Law, University of Kent, UK

**Keywords:** Traditional medicine, regulation, Mauritius, Ayurveda, technofutures, legalities

## Abstract

This article explores how traditional healing is regulated in the island of Mauritius. Drawing on postcolonial Science and Technology Studies and their encounter with socio-legal studies, it maps the emergence of the *Ayurveda and Other Traditional Medicines Act of 1990*, pointing out the selectivity of the notion of ‘tradition’ and its entanglement to broader nation-making processes, born in the process of its independence from Britain. While the social and political histories embedded in the text of the law favour the authorisation of some healing traditions and not others, outside its scope, other practices persist, and triggering competing visions about the island’s healing futures. By bringing into the frame the plurality of Mauritius’s healing landscape, where different traditions coexist despite their ambiguous legal status, this article accounts for the tensions in the regulation of traditional medicine, where the law produces its own inclusions and exclusions, and mundane legalities of everyday healing become sites of broader political questioning about the relationship between law, science, and nation-making.

## Introduction

This article explores how traditional healing is regulated in the Republic of Mauritius. It highlights the multiple layers that make up the legal framework of healing, weaving symbolic identity politics, everyday practices, and the pressures of international trade and politics. The paper takes as its starting point the most visible expression of the regulation of healing: the *Ayurvedic and Other Traditional Medicines Act of 1990 (*hereafter *Ayurveda Act)*. It emphasises the entanglement of the text in nationalism and identity politics, drawing the significance of this law as a vehicle to recognise Ayurvedic healing techniques used by the island’s Hindu-Mauritian population, and its significance to the governing elite of the time. Moving beyond this text, however, the everyday of healing constitutes a messy legal order. Different traditions coexist, including codified and non-codified knowledges attached to different identities represented within the island’s multicultural society. Aspects of these practices are valued in different ways by users, healers, and state agents. In a context where critiques have drawn attention to the complexity of identity politics in Mauritius, and the repeated erasing of creole identities from the public sphere, it is striking that this erasing also seems to be produced through the regulation of healing. Here, creole traditions are noticeably absent from regulatory discourses, despite their important role in the everyday. Drawing on Science and Technology Studies (STS), and their encounter with socio-legal studies, this article maps and explores this ambivalence, highlighting how the regulation of healing produces its own form of exclusion and inclusion through law, and how the apparently banal question of everyday healing becomes a site of broader political questioning.

Rather than the law surrounding traditional healing being a top-down process in which states impose a framework that healers follow, or being only a process in which law is permeated by social expectations of healing practices, the interface between law and traditional healing is dynamic and productive. State law embeds expectations, ideals, and visions of what role both tradition and science should play in the delivery of healing, and the shaping of state identity. Historical and institutional trajectories influence the techniques and approaches that states adopt, and the kind of role that different actors of healing can play formally and informally. Meanwhile, healing practices unfold against lines of legality and legitimacy, at times being restrained by regulatory demands, and at others diverging from formal expectations, following alternative normative orders instead. This is complicated in postcolonial contexts by the embedding of colonial histories in both legal and medical institutions that continue to create tensions between institutional and social identities.

Part of a broader project, our case study in Mauritius aimed to explore what the regulation of healing might mean in a country where questions of diversity and representation unfold in a space characterised by the absence of a distinct native population, where colonial and postcolonial trajectories are particularly layered, and both the exceptional biodiversity and cultural plurality have contributed to the building of the independent nation. In this article, we sketch the overlapping legalities against which healing unfolds, and focus on the ambivalence embedded in relevant state law, and its disconnection from everyday practice. We emphasise the tacit exclusions in the legal landscape, offering a reflection on broader questions over the place of the Creole population in Mauritius, and limited attention given to traditions relying on local healing plants. Instead, these traditions coexist unofficially with practices that have been granted a different kind of protection, while the potential of local biodiversity is reinscribed within anticipatory discourses turned more towards science than towards tradition.

We explore the relationship of law and healing through three distinct questions: First, we highlight the ambivalence embedded within the core of the *Ayurveda Act*, as it offered formal recognition to only three forms of traditional medicine systems but left out those that formed in this originally uninhabited island. The effect was to draw a line between approaches formally recognised by the state and others, also feeding into broader identity struggles developed after its independence from Great Britain in 1968. Second, we interrogate how the practices that have been left out of the legislation operate in everyday spaces, away from the state, and on the border of il/legality, yet are tolerated and constitutive of broader representations of Mauritius as a multicultural state. Finally, we explore how medicinal plant knowledges have also been given renewed attention in public discourses since the 1990s. Much attention to plants has, however, focused on their role as biological and chemical materials, leaving aside the historical and socio-cultural connections that attached them to localised knowledge systems. Even though medicinal plants are expected to become agents of a technoscientific future aligned to regional and global pharmaceutical regulatory strategies, they also perform another imagined future, propelled by competing post-independence discourses that reimagine the island’s creole social histories and healing practices as an anchor for a global and outward-looking Mauritian national identity.

## Methodology

This article is part of a multi-sited research project exploring the regulation of medicine across selected countries in Europe and Africa. We have explored the expressions and trajectories of regulation across England, France, Ghana, Senegal, Mauritius, and the French department of La Reunion, charting the frictions that emerge in the regulation of alternative and/or traditional healing.^1^ In Mauritius, as elsewhere, popular healing practices and products were side-lined from the formal healthcare system during the colonial era. Today, some new relationships have been institutionalised and legitimised through law. However, institutionalisation has been selective and has discreetly set aside some of Mauritius’ longstanding medical pluralism. Where healing practices have not been formally regulated, their everyday expression is negotiated through other forms of legitimacy and ordering. Meanwhile, a modernising discourse emerged on the sidelines, one that sees medicinal plants as carrying a promising technoscientific future for Mauritius.

To explore these issues, our empirical work is based on a combination of interviews (conducted in English or French), ethnographic observations, documentary and archival research conducted in Mauritius in August 2018 and November and December 2019. Documentary and archival research were used primarily to understand the history and evolution of the *Ayurveda Act*, drawing on documents from the National Archives Department in Port Louis- which is the main repository of the nation's public records- as well as other public documents collected from the Mauritian government website. The electronic documents analysed referred to more recent debates (from 2000-2022) at the Mauritian National Assembly, as well as relevant laws and regulations adopted since the adoption of the Act. Other reports and white papers from the Ministry of Health and Quality of Life, cabinet decisions from the Prime Minister's office, and official press releases supplemented our analysis of the contemporary policies around traditional medicine pursued by the government. Our search criteria across these different databases included basic keywords like ‘traditional medicine’, ‘ayurveda’, ‘traditional Chinese medicine’, ‘herbal medicine,’ and ‘acupuncture.’

Alongside this, and to better understand the everyday legalities of traditional healing, we conducted 15 qualitative interviews (recorded and transcribed) with representatives of traditional medicine charities and associations, individual healers, and wellness providers. Although a second trip had been planned to collect further data, travel limitations during the COVID-19 pandemic prevented the latter. For that reason, we sought out additional information about the different traditional practices and discourses around them on Mauritian news media (primarily *Le Mauricien*, *L’Express*, *Defimedia*), some relevant international news releases, and publicly available information (websites, biographies, videos, press releases on social media) about the actors involved in the life of Mauritius’ traditional and alternative medicine milieu, such as politicians, charities, private companies, and other service providers.

## Approaching traditional medicine

The guiding framework of this article is located at the intersection between STS and socio-legal studies, seeking to explore the co-constitution of medical practices and legal relationships, both in their symbolic dimensions and in everyday practice. It also builds on a rich body of research that has explored the contemporary workings of traditional healing, and its constant redefinition, including in its interactions with the state.^2^ While the regulation of traditional healing organises pasts and cultural histories, it has also been mobilised to negotiate future identities, of both states and communities.^3^ In other words, ‘traditional medicine’ can be about preservation as well as reinvention, and it often sits at a crossroads.^4^ These transformations can be the product of ‘top-down’ or ‘bottom-up’ processes which are tangled up to different temporalities, and sticky processes of redefining the interaction between biomedicine and traditional medicine in postcolonial states.^5^ Indeed, much of the impetus behind the regulation has been driven by the desire to redefine the space and value of traditional and alternative systems in the context of decolonisation. Part of this task has involved reversing the branding of indigenous knowledges as ‘backward’ or ‘irrational,’ a tactic which had cemented the socio-political power of colonial authorities by producing new objects of governance and persecution.^6^ The undoing of these histories has also called for a re-evaluation of the role of subaltern actors in the process of science-making during colonialism and their place in the history of science. Indeed, while European colonialism imposed its legal and biomedical logics across territories, it also appropriated or relied on subaltern and indigenous healing practices as and when it suited its needs.^7^ However, their legacy remained largely unrecognised, and the deployment of rationality as an essential characteristic of scientific endeavours demarcated Western knowledges and technologies as incommensurable from those found in the colonised territories. More recent historical research has nevertheless shown how these power dynamics were more nuanced, and how necessity forged complex relations of interdependency where subaltern actors forged their own trajectories. In Mauritius, for example, enslaved Africans did not just develop ‘know-how’ about the cultivation of medicinal plants, but developed botanical knowledge which was shared to negotiate their social status.^8^

Although contributions like these were erased in the historiography of botanical sciences, subsequent political and legal struggles to decolonise public health set the stage for postcolonial governments and movements to embrace traditional medicine as a powerful symbol of nationhood, economic development, and pharmaceutical sovereignty.^9^ Throughout the 1960s and 1970s, the World Health Organisation (WHO) became the platform where these aspirations were articulated in key political resolutions, and in a radical definition of traditional medicine spearheaded by African delegates, which recognised the value of tacit and experiential knowledges transmitted across time.^10^ However, from the 1990s onwards, the work by the WHO underwent a technical turn which aligned traditional medicine much closer to biomedical legal frameworks and conditioned their integration on becoming more scientifically-shaped.^11^ This transformation responded, in part, to the hopes and expectations that governments of the twentieth century have placed on science, industrialisation, and medical technology, as catalysts for modernisation and economic development.^12^ These expectations have been compounded by the rise of global pharmaceutical markets and the opportunities presented by the disenchantment with modern medicine in Western countries.^13^ Aware of the risks and opportunities presented by the globalisation of traditional medicines, governments have conditioned their legalisation to the ‘professionalisation’ of their knowledge, and the transformation of traditional medicine products into pharmaceutical forms (such as pills).^14^ However, these regimes do more than legalise an existing practice, as they translate, translocate and transform traditional medicines^15^ by entangling them to risk governance mechanisms^16^ and to ‘pharmaceutical assemblages’ that are transnational in nature.^17^

Stakeholders, whether it is healers, professional associations or the state, adapt to these pressures yet confront emerging regulatory demands in their own terms. Where a cultural identity is deemed to be at risk of being excluded from, or incorporated by dominant medical knowledge systems, actors have doubled down on the radical alterity of their medicines by reaffirming a particular national, cultural, or regional identity.^18^ Others have strategically reframed traditional medicines along different temporalities, or what Subramaniam calls ‘archaic modernities.’^19^ Here, the spiritual and cultural elements of traditional medicines are not extracted, but exalted and the relationship with Western science is reversed: if science has not yet understood the mechanism of action of these therapies, it is because it is not advanced enough to understand them. For example, nationalist governments in India have revived and reframed Ayurveda as being already thoroughly scientific, and the Ayurvedic industry as a pillar in its plans to project the Indian nation as a global technoscientific power.^20^ Such ambition has played a determinant role in shaping the place of Ayurveda worldwide, among Indian diasporas, including those in Mauritius.

How particular states choose to orient themselves in relation to science and technology, and draw the boundaries of what constitutes scientific or technological futures, generate performances that shape political institutions and social opportunities.^21^ Similarly, which practices and bodies of knowledge attract the interest of the contemporary state, and how they are framed against its imagined modernity, has significant implications for the relative position of different groups in public life. As noted by Langwick, the ‘reinvention’ of tradition, produces ‘contradictory and partial projections’ of the public, embodying different political projects and promises.^22^ Indeed, the process of legitimising traditional medicines is far from simple as it can become an ‘uneven and contested process.’^23^ Here, stakeholders hold competing visions and problematizations about unregulated healing practices and expect the state to bring order by reorganising the healing landscape through the integration of traditional medicine into national and global health regulations, systems and economies. Thus, if the politics of traditional medicine is a contested field, the law is sought out for its believed ability to organise, translate and solidify normative boundaries, or even enact new subjectivities and national futures.^24^ For this reason, the law plays a key role in embodying these orientations, both symbolically, and in shaping the possibilities or limitations of different forms of practice and healing materials. In doing so, it not only ‘reinvents’ traditions, but it transforms and reorganises the ways people relate to healing, by drawing new lines of legality and illegality.

At the same time, the ability of the law to capture and determine the future place of healing traditions is limited by everyday practices, where individuals and communities draw their own preferences, and adapt to ever-shifting realities.^25^ This produces a multiplicity of medical traditions and legalities: informal legal orders co-exist alongside the formal systems created by states, enacting lines of legitimacy that are juxtaposed to the formal legal system.^26^ The effects of legal norms are decentred and often unpredictable, shaping in direct and indirect ways the boundaries of legitimate knowledge, and creating lines of visibility and precarity between claims and practices. Because legal status does not completely determine the reach of particular practices, healing techniques that lack the formal assent of the state often thrive in their own way. These layered and often overlapping relationships between legalities and practices are visible in the Mauritian context, where the legal system celebrates pluralism in healing, yet renders others unacknowledged. Those left behind or at the margins of the law are not deterred, and instead, adapt by relating with and embedding aspects of the legal frameworks to their own practice.

We explore these issues in three parts: First, we map the emergence and significance of the *Ayurveda Act* and its role in reorganising what had been a fluid and plural field of traditional healing practices. In this section, we note how, instead of legitimising old practices, the law mirrors biomedical norms while enacting new ones for the traditional medicine systems recognised in the law. The law can be read as part of a postcolonial nation-building project specific to Mauritius and as a response to support the global ambitions that India has projected for Ayurvedic medicine. Second, we turn to the contrast between the selective form of pluralism adopted by the law, and the diversity and co-mingling of healing practices that characterise Mauritius. Here, we discuss how the law has produced new layers of legality and illegality by privileging the visibility and potential futures of some healing traditions over others.^27^ Across the board, actors deploy their own reading of law, both in its symbolic stakes and material implications, layering the effects of law with their own ‘interpretive framework and a set of resources with which and through which the social world (including that part known as law) is constituted.’^28^ Finally, we explore how a different vision for the future of traditional medicine emerged alongside that offered in the *Ayurveda Act*, calling for their own forms of regulation. Prompted by specific political configurations, medicinal plants native to Mauritius have been positioned as a potential route to a different kind of African healing future for Mauritius.

## Ayurveda diplomacy and traditional modernities

This section contextualises and analyses the genesis of the *Ayurveda Act*, paying attention to the social and political configurations that framed its adoption and the different arguments that unfolded. These debates show how the drive to officialise Traditional Medicine was strongly linked to the reconstitution of an Indian identity, explaining the prevalent positioning of Ayurvedic medicine over both homoeopathy and traditional Chinese medicine (the ‘Other Traditional Medicines’ also included in the law), in the phrasing and titling of the Act. The Act grants an ambivalent recognition of Traditional Medicine, which it ultimately defines in specific and narrow terms, given the medical pluralism that defined Mauritian health practices at the time and continues to do so.^29^

Some context is helpful to understand the stakes of the debates over the Act. The Mauritian political and legal system has been shaped in part by its colonial history, and the logics of both the British and French states persist in its institutions and laws, including in the provision of healthcare and laws governing medicine. Before independence, colonial medicine in Mauritius had been centralised under the Chief Medical Officer’s authority, and medical practitioners, as well as education, had been sourced from Britain and other colonies. Although the colonial healthcare system has been largely overhauled, and today’s public healthcare system relies on the provision of universal healthcare, the legal and healthcare system still embed traces of colonial history, and of both French and British laws.

When independence from Britain became increasingly likely after the 1940s, different segments of the population feared Indo-Mauritian control over the new national government due to their demographic majority.^30^ These fears resulted in the institutionalisation of communal politics and a ‘Best Loser System,’ that was meant to guarantee the representation of minority ethnic groups in the Legislative Assembly (today called National Assembly). Based on the ordering of identities that were used in the colonial census (Hindus, Muslims, Chinese, and General Population),^31^ This electoral system is credited for the absence of interethnic conflict in Mauritius, often deemed a ‘rainbow’ nation for its prosperous multi-ethnic society.^32^ Yet, a side effect of this system is that the constitution entrenched communal identities to the detriment of Mauritian creoles, who were lumped with Franco-Mauritians under the category of ‘general population.’^33^ The search for rootedness in Mauritius’ ancestors, and the political distribution of power that has entrenched these social arrangements, have been detrimental to those who have no singular identifiable ancestral origin, or whose origins tie them specifically to former slaves.^34^ This is the case for Creoles, Afro-Mauritians that descended from the slaves brought from Madagascar and Eastern Africa in the seventeenth century.

The *Ayurveda Act* of 1990 reproduced such societal tensions around identity politics, and their expression through both symbolism and practice. From the outset, the significance of this law lies in its ambitious reach to cement a material and symbolic relationship of the dominant political and cultural group with India through a law that legalised the practice of Ayurveda. Attachments to Hindu identities run deep in the genesis of the law. To begin, the drafting of the bill itself had been an initiative proposed by a well-known Hindu charity, the Human Service Trust (hereafter HST), and a New Delhi Ayurvedic doctor, Pandit Jugdish Sharma. Keen to open centres of treatment but aware of the legal ambiguity around Ayurvedic medicine, and the absence of a professionalised body of Ayurvedic doctors, the Board of the HST requested the government to draft a law to legalise their practice and enable them to bring Indian doctors.^35^ Of course, traditional medicine practices associated with South Asian medical traditions, preceded the establishment of the charity but the HST played a central role in its institutionalisation and legitimisation. They had privileged access to the government ever since its founding father, Swami Krishanand, and the centre itself, had become a symbol of Mauritius and India’s shared paths of independence from Britain. This history made it a prominent institution among high-ranking Indo-Mauritian politicians who were warm to the idea of institutionalising and integrating Ayurveda into public health care. This included none other than Anerood Jagnauth, the incumbent Prime Minister who served his first term between 1982-1992, and Shri Parmanand Ramlackhan, a public figure associated with the Trust, and promoter of integrating Hindu culture- both of whom were keen to establish a closer relationship with India.^36^

These connections did not go unnoticed during the second reading of the bill, where some legislators expressed scepticism about the utility and purpose of the law. Although the bill was widely welcomed because it would finally recognise the influence of Indian culture on Mauritians, some representatives expressed doubts. For example, Dr Boolell, a medical doctor cautioned against embracing this new law uncritically and argued that the law was being used as a tool to strengthen bilateral ties with India by advancing Ayurvedic medicine in Mauritius. Others were very explicit about the national histories that prompted the desire to embrace Ayurveda. For the leader of the opposition, Dr Nababsing, the colonial suppression of the medicine of Mauritius’ forefathers warranted their restoration in the newly independent state, and anyone who opposed it had internalised colonial rule. Such strongly anticolonial interventions flesh out the symbolic functions attached to the law: the legitimation of Ayurveda actualised a desire to rupture with the past, including the trauma of indenture and everything associated with it, such as the suppression of healing practices that had been branded as superstition.^37^

The inscription of Chinese medicine and homoeopathy within the law was secondary to the debates, a logical consequence of opening up the recognition of traditional medicine to other institutionalised systems of practice, while also creating other opportunities for international trade and cooperation.^38^ However, the title of the Act itself enunciates a particular ordering of traditional medicine, that echoes a broader state’s privileging of Ayurveda in public healthcare policy.^39^ For all three practices nonetheless, the legal framework offered by the *Ayurveda Act* reproduced a well-known blueprint in the regulation of medical and health professions: it legalised traditional medicine practices; and created a Board composed of both biomedical and traditional medicine experts entrusted with the task of administering, monitoring and penalising members in the national register; created conditions of practice; and made it illegal for unregistered persons to perform any act of traditional medicine.^40^ As we return to it, the latter meant that only those who practised Ayurveda, Chinese Medicine or Homeopathy could claim to legal practice ‘traditional medicine,’ and that such a claim was not possible for those who practised other healing techniques.

Despite its ambitions, the effect of this legal framework in practice is questionable. It has been at its most successful in supporting the institutionalisation of Ayurveda into the public health system, facilitating in turn bilateral cooperation with India, and to a lesser extent, with China.^41^ For example, there are six Ayurvedic hospitals, mostly overseen by doctors from Mauritius who have qualified in India or Indian doctors whose qualifications have been recognised by the Mauritius Ministry of Health. Bilateral cooperation between India and Mauritius grew stronger after 2016 when both countries signed a memorandum of understanding for greater cooperation in the form of mutual recognition of their traditional medicine systems, supply of traditional medicine substances, mostly from India to Mauritius, and a research collaboration scheme with Mauritius’ new research centre. Other initiatives include a long-established program of scholarships to study in India and become qualified as Ayurvedic doctors, cultural celebration days (such as Yoga and Ayurveda Day), and during the pandemic, donations of Ayurvedic medicine by the Indian government.^42^ State hospitals tend to follow the requirements set out in the law, and Ayurvedic practitioners there are licensed accordingly.

However, the distribution and dispensing of products in pharmacies are less effectively regulated at the many retail shops in the country. In contrast to the strict regulation of practitioners, there is no equivalent for traditional medicine pharmacists and retailers, and those who sell pre-packaged imported traditional medicine products (mostly classic and propriety Ayurvedic and Chinese medicines) do it without a prescription by a registered practitioner. Although traditional medicine products also fall outside of the scope of pharmaceutical regulations,^43^ clearance is granted by a dedicated Ayurvedic Committee under the Ministry of Health and Quality of Life. The requirements for certification set by the Committee include, among others, a list of all individual ingredients in the product, a certificate of microbial and heavy metal analysis, Good Manufacturing Practices (GMP), and the endorsement by a registered Mauritian traditional medicine practitioner.^44^ Even though traditional medicine products are yet to be regulated by a specific law,^45^ their inclusion within public healthcare, under the stewardship of Ayurvedic experts, highlights how the legitimation of traditional medicine becomes a highly symbolic act that not only privileges ‘Ayurveda’ as a medical system within government structures but also one that materialises specific relationships and national futures.

## From symbolic legislation to everyday healing

As noted, the legislation defines the scope of traditional medicine to englobe only certain practices to be conducted by registered practitioners and prohibits the performance of any acts of traditional medicine by lay persons. Yet, in this dimension as well, the law’s effects are questionable as everyday healing in Mauritius is grounded in a much wider range of knowledges, materials, and techniques than those listed in the Act. This includes aspects of the traditions brought by South Asian and Chinese immigrants,^46^ that were blended with other healing practices, including the existing tacit knowledges of the use of endemic and native medicinal plants. Whether used on their own or embedded in rituals rooted in different religions and cultures, different ethnic groups in Mauritius have been found to use similar local medicinal plants.^47^ However, during the debates of the Ayurveda Bill, some of these tacit knowledges were repudiated, while others were discussed, but only as an afterthought. Instead, on the few occasions they were mentioned, including those involving rituals and sorcery or sometimes referred to as black magic or voodoo, their significance was dismissed as not truly belonging to the type of state process that the legislation was concerned with. As one assembly member noted, ‘priest physicians or *traitere*,’ that relied on ‘bringing black cocks, goats and sheep for supposedly sacrifices’^48^ were superstitious or retrograde practices.^49^ Such repudiation is not uncommon: where other states have legislated on traditional healing, spiritual elements are often seen as something that needs to be left out of the scope of legal practice.^50^

The dismissal of the island's tacit knowledges of medicinal plants was even more striking, given the significance of Mauritius as a biodiversity hotspot with a high concentration of native and endemic plants. Indeed, some of these knowledges have been traced back to Malagasy and African slaves who helped cultivate and acclimatise plants from across imperial territories and developed their own knowledge of the medicinal uses of native plants.^51^ While these ethnobotanical practices grew locally, they were further shaped and transformed by their mingling with other medical traditions, including those from Africa, Europe, China, and the Indian subcontinent.^52^ This resulted in a collage of medical beliefs, where each reverberation has been the product of the continuous integration and mixture of medical beliefs brought by different migration waves. Women were also at the heart of the transmission and transformation of these herbal knowledges. Whether as matrons or nurses, their lives and roles crossed the social boundaries between masters and slaves, and it is at these crossroads where different knowledges syncretised.^53^ And while colonial actors, from botanists to doctors, adopted and mobilised subaltern knowledges about plants, their contributions ‘disappeared […] or never entered the European accounts of the time.’^54^ Instead, the local informants who shared their knowledge with European botanists, the authors of the first books documenting medicinal uses of plants in Mauritius in the mid and late nineteenth century, have been consigned to anonymity.^55^

Today, these tacit knowledges, based largely on oral transmission, have been dispersed across different practices and they manifest different political and epistemological orientations that map onto existing tensions around multiculturalism, and on different understandings about creolisation.^56^ For example, in her 1970s research on Mauritius medical beliefs, Sussman described ‘creole’ healers as town herbalists or healers of black African heritage, who combine different perspectives and healing resources, using medicinal plants on their own, and as part of ritualistic healing practices. More recently, projects trying to revive the use of medicinal plants have made direct reference to this term, as we will turn to later. Yet others reference creolisation indirectly, as an idea burrowed within the subtext of new therapeutic practices and in their framing as being ‘naturally’ Mauritian. These claims are made through a chain of associations between symbols: the island’s biodiverse landscape and the rainbow as a representation of Mauritius’ multicultural national identity. If these associations become a ‘performative claim of belongings,’^57^ it is because they are grounded in the island’s history of medical eclecticism, and in *creolisation*, understood as a process where Mauritius is the ‘product of multiple influences, multiple sources, which to differing degrees merge, take root and naturalize.’^58^ As one of our informants argued, the healing knowledges from China, India, Africa and Madagascar were embedded in Mauritians’ consciousness: ‘We have it in our genes, the knowledge that we use nature medicine, nature as medicine.’ In practice, these ideas have been translated into a mixing of medical beliefs and techniques (flower essences, acupuncture, osteopathy, energy healing, etc.), catering to foreign tourists and middle- and upper-class Mauritian customers at wellness centres in high-end hotels or in private practice. While not expressly labelled ‘creole’ or as ‘traditional’ medicine, these meanings are elicited in the blending of approaches, and in the projection of Mauritian hybridity. As one informant explained, ‘the uniqueness of Mauritius is the blend,’^59^ a view echoed in an anecdote by his collaborator, a wellness designer, about how they conceptualise their services: ‘I said to him, let’s create a Mauritius massage. We are going to blend all your knowledge because the massage in Mauritius is not like the pure massage from India. It’s a blend.’^60^

What is evident in the debates and the outcome of the Ayurveda Bill is that the island’s eclectic healing traditions were filtered and re-organised: codified medical systems were favoured by the state, while plant knowledges, especially those used alongside ‘superstitious’ rituals, were seen as too dispersed and anecdotal to have enough legitimacy. The Act’s recognition of codified systems may well be rooted in the well-defined and structured body of knowledge about human physiology, pharmacology and pharmaceuticals, which facilitates their integration into existing regulatory regimes modelled around biomedicine.^61^ By contrast, the oral transmission of indigenous and folk healing practices have been lost in time, and while often revitalised through their integration into codified systems, elements that were part of these ‘traditions’ are lost in the process of translation: vernacular plant names, prayers, and other rituals are side-lined, sometimes as a condition to be legitimated, particularly through law.^62^ This is evident in some exchanges during the debate, where legislators shared stories about their use of medicinal plants, yet these stand out on their own, without a reference to the rituals and healing cultures attached to them. At the same time, these exchanges show how engrained medicinal plants have been in Mauritius, and how legislators were aware of their significance. To illustrate this, Ruhee pointed out that the government was not paying enough attention to what Mauritius's biodiverse ecosystems had to offer to the advancement of ethnopharmacology. In response, other legislators volunteered a list of names of the different plants they knew had healing properties: *Liane*, *Baume du Perou*, and *Saponaire* (also known as periwinkle). Meanwhile, Duval reminded the Assembly that a group of French scientists from the *Agence du Cooperation Culturelle* had come to the island to research plants, and the government should locate the report about their visit. Others responded by stressing the need to protect these resources from indiscriminate exploitation.

Despite the awareness about the use of plants in Mauritius, and the fact that the so-called ‘craze’ for herbal products was one of the justifications for bringing a traditional medicine law forward,^63^ the Act itself is silent about their regulation, or the value to be given to healers who rely on tacit knowledges that use medicinal plants. In this tacit exclusion of Mauritius tacit medical cultures, which are themselves the product of the circulation, transfer and exchange of local and foreign knowledges, the law also reflects a broader pattern in state symbolic decisions, where Creole identities, both understood as those derived from African histories, and as mixed identities, are excluded or side-lined in legislative or policy decisions.^64^ The legitimation of identifiable ‘traditions’ associated with the governing Hindu-Mauritian parties, at the exclusion of the local heterogeneous healing knowledges, underscores what Mukharji characterises as the tendency to reduce subaltern therapeutics ‘as limited “versions” of elite systems.’^65^ In Mauritius, as in India, therapeutics that defy classification are either absorbed or discarded by the process of ‘ayurvedicalisation,’ flattening existing therapeutic pluralities through the institutionalisation of ‘nested’ hierarchies of knowledge.’ Indeed, the Act enabled particular versions of Ayurveda to be given a very visible role within the state infrastructure, while other systems became subordinated (such as Chinese medicine) or completely excluded from. To the extent that the eclecticism embodied by Mauritius’ healing cultures defied systems classifications, they became ‘relegated to the status of folklore “localism,”’^66^ persisting at a relative distance from the law.

This exclusion from the text of the law has not stopped the existence of these practices. To the contrary, many continue to thrive and provide everyday care in a way that is both highly visible and deeply embedded in local cultures. But the fact that the text specifies that only those registered, according to the law, should be allowed to practice under the label of ‘traditional medicine,’ and that such registration is only opened to the three named traditions, means that many healers are left to practice in a grey legal space. This includes many practices that are specific to Mauritius and a range of more recently complementary and alternative healing techniques such as the ones illustrated earlier in this section. While conversations on the latter are a rather separate matter in policy discourses, they share the same legal fragility as far as practice is concerned. Users and practitioners relate to this uncertain legality in different ways, some coexisting with limited concern for it, seeing legal recognition as desirable but its absence irrelevant to their practice, while others seek to subvert or contest legal boundaries out of a belief that the existing law is unjust.^67^

Unregulated healers experience different levels of precarity (depending for example on the type of products they rely upon, or how much their work may overlap with Ayurveda). Some are acutely aware that they work (in the words of one healer) ‘actually illegally’ because the label of ‘traditional medicine’ is strictly regulated. Years of demands and negotiations have not so far led to any changes to the law to integrate a more open-ended set of healing practices. The remaining uncertainty and lack of clarity over which rules apply to specific practices was an issue for some of our informants:

The thing in Mauritius with the law, we don't have a real law stating that you must be a practitioner for so many years and be able to deliver your practice from your centre. In general, we should have had the law, but it's not very clear. It's not very clear about which practice to practice and what are the different rules and regulations to follow and not to follow.^68^

Others express less concern and do not see the law as it is as detrimental (nor immediately relevant) to their everyday practice. This is partly because they appeal to a more holistic approach to individuals’ ‘wellbeing’ and regard their blended therapies as being less centrally ‘medical.’ Even though their experience has not been interrupted, or challenged by the state, those who combined different therapies were the ones most concerned about the potential scenarios where state authorities would overreact to new therapies. As one informant noted, ‘if one day something is going wrong or someone wakes up and thinks that we should follow the regulations of this country, it could stop our activities.’^69^ To date, these healers operate openly as licensed businesses, and because some of them have delivered training, they have had to register with the Mauritian Qualifications Authority. By avoiding making claims about their medical identity, and instead, embracing hybridity, they have side-stepped the regulatory demands of the Ayurveda Act.

Meanwhile, those more directly dependent on medicinal plants in their everyday lives have experienced different kinds of friction with the law. For one of our informants, a creole ‘shaman’ who descended from Mozambican slaves and Indian indentured labourers, land access is crucial to him and his community due to their reliance on medicinal plants foraged around a basaltic monolith at the edge of the island’s Southwest coast. However, rapid population growth and deforestation for agricultural development have decimated Mauritius’ forests, radically shrinking the land where precious endemic and native flora can thrive.^70^ For communities like these, access to plants has been compounded by the legacies of colonialism: Originally evicted by the French landed gentry of the area, the villagers are now under pressure from the development of the area into a heritage and eco-tourism hotspot promoting spiritual ‘wellness’ therapies.^71^ However, recognition of Le Morne as a site of cultural, spiritual, and material significance for Mauritian Creoles has given some reprieve from these pressures, and a lifeline to the mountain. As our informant explained, the mountain represents a sacred place where he listens to the spirits of plants, and the stories of pain of his ancestors, who are said to have lived around here, after escaping the sugar plantations. Because these histories reverberate in his everyday practice, and his knowledge is a spiritual calling, he refuses to commercialise medicinal plants and regards their sale as the exploitation of a living being.^72^

Although few herbalists buy plants for their practice, not all herbalists share the same reluctance to sell plants, and stalls selling herbal remedies are commonplace in the capital, Port Louis. A prominent herbalist we spoke to began working in his father's stand at the local market when he was only seven: ‘For us, it’s coming to the market to learn the herbs, the name of the herbs, the vernacular name, the local name of the herbs.’ His ancestors are Tamils from Southeast India, and he explains they learned to use plants in Mauritius that were like those in India. He possesses a combination of skills acquired from a truncated qualification in pharmacy and knowledge inherited from family members who’ve traditionally worked as *herboristes* (usually male members). The market stall is regularly busy, with customers from all over the island. He also ships teas to international clients, but these are exported as food products. Because he regards his knowledge as highly specialised, he strongly supports efforts to improve the quality of herbal products and regards sellers with limited knowledge about plants as obvious risks. Although both his expertise and his products fall outside of the scope of the Ayurveda Act, he interacts and cooperates regularly with state authorities, sharing his experience as a local herbalist at international conferences, or in the regular submission of his teas to the agricultural authorities before their exportation.^73^

Across the island, other initiatives have sought to maintain the knowledges over local plants, sometimes adapting them to new demands or imperatives. For example, a recent development initiative, named ‘Secret de Grand-Mère,’ or ‘Grandmother’s Secrets’ sought to reclaim women’s less visible role in the cultivation, production, and transmission of healing plants in Mauritius. When the program struggled to sell plants in the raw, it adapted to contemporary consumer behaviours by selling mixtures of teas in modern packaging.^74^ While rescuing tacit herbal knowledges was not the main objective of this project serendipitously or not, it tapped into emerging political narratives that exalted the appeal of plant medicines and the conservation of the island's biodiversity, some of which were also recaptured in a different techno-political project we turn to next.

## African phytotherapy: laboratories of hope

If these tacit knowledges were not given direct attention within the *Ayurveda Act*, and today they operate largely aside from any formal state intervention, the plants it relies on have gradually become the object of more explicit scientific attention and regulatory aspirations. In this newer mobilisation, plants have been positioned in the discourse as untapped materials that represent the distinctive identity of Mauritius. Their value as healing materials is to be harnessed, through the proceedings of science, and the modes of recognition attached to the scientific-industrial complex. This possibility is conditional on their ethnobotanical identification, classification, and the creation of suitable regulatory regimes that would advance ethno-pharmacological research in the future. Yet this trust in science to produce opportunities for the diffusion of Mauritian healing also risks obscuring the forms of tacit knowledges that foster medicinal plant use.

The increased focus on medicinal plants as scientific resources in public discourse was propelled in part by the interventions of local scientists, including most prominently former President of Mauritius, Ameena Gurib-Fakim, a Muslim Mauritian who obtained a PhD in Chemistry in the UK. Her presidency was characterised by her drive to internationalise Mauritius medicinal plants by placing them at the centre of the country’s scientific and technological development. More importantly, and in contrast to the *Ayurveda Act*, which had emphasised the Asian roots of Mauritian medical traditions, her vision about the future of Mauritius medicinal plants was tied to an African ‘geographical imaginary.’^75^

Scientific attention to the island’s medicinal plants began to take shape in the late twentieth century, almost at the same time as the Legislative Council debated and approved the *Ayurveda Act*. There had been efforts to document the island’s botanical medicine resources earlier, but they were sparse, even though biodiversity loss and the growing role of biomedicine in public health were increasingly contributing to the gradual loss of medicinal plants and the knowledges around them.^76^ By the 1980s, local scientists funded by regional and international bodies, spearheaded efforts to codify and preserve the island’s ethnobotanical resources. Under the aegis of the Indian Ocean Commission and in partnership with the Mauritius Herbarium, scientists at the University of Mauritius (including Gurib-Fakim), identified over 600 locally used medicinal plants using data from surveys collecting knowledge about the uses of medicinal plants throughout Mauritius and the island of Rodrigues, supplemented with laboratory analysis.^77^ This project, which culminated in the publication of a compendium in 1994, was mired by political controversy after the Mauritius Sugar Industry Research Institute, which houses the Mauritius Herbarium, claimed ‘ownership of plant-related work.’^78^

From the early 2000s, this research moved from such inventory towards a more commercial enterprise with a French investor.^79^ This included, among others, the development of patentable products for the food and cosmetic industry based on medicinal plants. While being herself involved in such endeavours, Gurib-Fakim was also involved in developing quality standards for the African region, through the African Medicinal Standards Association (AMSA),^80^ which culminated in the publication of the first comprehensive African Herbal Pharmacopeia, aimed at unlocking barriers for trading medicinal plants in international markets.^81^ The unlocking of an industry based on Mauritius endemic medicinal plants hinged also on the adoption of the Clinical Trials Act of 2011, a legislation which in her view could transform Mauritius into a regional pharmaceutical and clinical research hub because of the genetic diversity of its population.^82^

Her role as a spokesperson for medicinal plants was further amplified after becoming the president of Mauritius in 2015. Positioning herself as a ‘scientist president’ across different national, regional, and international networks, her discourses promoted the transformation of Mauritius medicinal plants into pharmaceutical and cosmetic products through adequate regulation. Indeed, much of this scientific and legal activism over the years had been partly born in her frustrations about the lack of a proper ecosystem in Mauritius for the protection of academic intellectual property that she experienced as a researcher and an entrepreneur, and her goal was to enable the convergence of science and capital. To that end, her statements consistently framed Mauritius’ native and endemic flora as an engine for the development of Mauritius, and Africa more generally, treading carefully around the fragility of its biodiversity, and the mandates emerging from international legal frameworks on biodiversity conservation, intellectual property and benefit sharing:^83^

With the appropriate infrastructure — technical, legal, and regulatory — this treasure trove could translate into enormous wealth. In my view, this would create opportunities for Africa’s youth. I have been laying the groundwork for that translation as an academic, documenting uses of medicinal plants, as an entrepreneur and, most recently, as president of Mauritius. (…) I believe key to both tasks is our unique biodiversity.^84^

While statements like this show how her position embodied broader tensions at the crossroads of private and public interests in global intellectual property and bioprospecting practices alike, this entanglement contributed to the ambivalent trajectory of medicinal plants in Mauritius. On one hand, this framing conditioned the development of Mauritius’ ‘green gold’ to the strengthening of technical capabilities and the adoption of pharmacological and biomedical regulatory frameworks, including regulations to conduct clinical trials, enable the standardisation of extracts, and facilitate their manufacture and distribution in international markets. Yet, by conditioning the value of plants on their transformation through scientific processes, these discourses performed a ‘domaining’^85^ of medicinal plants, where the latter were analytically separated from their past social ramifications, including their historical discovery, use and preservation through forms of knowledges other than scientific. If these discourses focused on developing an industry where scientific research and investment in plants could be patented, they were also deeply contradictory as they both exalted and glossed over Mauritius’ hybrid origins. In doing so, complex questions about knowledge ownership and benefit-sharing have been avoided or postponed.

Finally, even though her legal activism did not materialise in the drafting of national laws or guidelines relevant to the regulation of plant-based medicines, they are relevant ‘anticipatory discourses.’^86^ As such, these discourses mobilised new normative orientations and new modes of relation with medicinal plants through appeals to international and regional governance instruments and mechanisms, such as the WHO and the World Intellectual Property Organisation (WIPO).^87^ These appeals also elucidated the role of the law in the generation of new orientations and possibilities for Mauritius’ collective prosperity, facilitated through research and investment, both essential tools towards the materialisation of Mauritius as a living laboratory. More broadly, these anticipatory discourses represent a competing vision for Mauritius future, one that engineers a future that is not tied to Indian globalisation, as scripted by the Ayurveda Act. Instead, the entanglement of ethnomedicines to African and global imaginaries of scientific progress embodied another kind of performative claim of belonging, one that also filtered and reorganised Mauritius healing histories by pointing firmly to a modern African future.

## Conclusion

Overall, in the context of Mauritius, the regulation of traditional medicines and the legalities that emerge alongside illustrate the broader politics and postcolonial trajectories that underpin the regulation of healing. Exposing the redefinition of traditional healing by elite social groups and their transformation into national medicines, this article has questioned the relationships between Mauritius codified medicine systems and tacit knowledges, and the effect of the constitution in sustaining a particular legal ordering. Here, the *Ayurveda Act* offered a particular reading of what traditions the Mauritian state seeks to build upon and render visible.

On the one hand, the Act was based on an idea that traditional medicine could be institutionalised by the state in ways that had both symbolic, geopolitical and economic implications. On the other hand, it drew lines of legitimacy over the type of cultural heritage that would define traditional medicine as defined by the state. Because the proposed definition of traditional medicine was partial, and as seen in other contexts,^88^ a hierarchical organisation of healing emerged, whereby Ayurveda sat in a privileged position within the public healthcare system. Through this vision for traditional medicine, attached to a specific idea of how Mauritius’ post-independence modernity should look like, the law rewrote what had constituted until then a healing landscape that was intensely plural and fluid. While some of the island’s healing traditions continue to thrive, their legal position is fragile, especially for those who use techniques that overlap with codified systems and those relying on medicinal plants.

The impetus to harness the value of medicinal plants for scientific development may, at the surface level seem to engage other types of Mauritian heritage, more geographically embedded in the political imaginaries of the African region, and to discourses that revalue the island flora. Here, Mauritian scientists valorised traditional medicine but offered conditional support to local knowledges attached to plants. Under this framing, the survival of medicinal plants and their knowledges was premised on the shedding of their cultural and spiritual remnants, and their codification into distinct technical and scientific objects to be distributed on global pharmaceutical networks.^89^ Such distancing may be inevitable to become amenable to international regulations conjuring alternative futures and to spatial orientations and temporalities that are tied to the colonial and postcolonial struggles of the African and Asian continents.^90^

Detached from the creole histories underpinning their use, discourses surrounding the future of medicinal plants in Mauritius also sidelined the specific knowledges that had underpinned their use, sometimes explicitly playing down questions of heritage and sovereignty over genetic resources. For example, when considering the regulatory conundrums around sharing the benefits of commercialising the products of traditional knowledges, the former president appealed to the Creole histories of the island, claiming that the research company she had co-founded had a ‘big competitive advantage’ because the scientists researching the plants were natives themselves and that there were ‘no indigenous peoples.’^91^ Beyond the complex debates about intellectual property and benefit sharing of indigenous knowledges, this anecdote crystalises two types of elisions at work in the Mauritian context: first, the construction of ‘publicness’ in the formulation of traditional medicine laws in Africa, and more specifically, the exclusions of the continuous ambivalence underpinning creole identities. On one hand, the exclusion of Creole healing traditions mapped onto existing social scripts and political struggles where Creole identities symbolise non-belonging and ‘uprootedness’ while other ethnoreligious groups have been essentialised ‘old, deep, and rooted’ identities.^92^ While creoles have sought in recent years to contest these erasures through a reclamation and memorialisation of the histories stemming from African slavery, further tensions have emerged as middle-class Mauritians of mixed heritage have also contested the organisation of national identities around fragmented ethno-religious identities. Here, creolite has been re-signified as a new national identity that celebrates the islands’ histories of social plurality through the claim that creoles are the ‘only *vrais Mauriciens*,’ since they are the only group who, as it were, emerged from the Mauritian soil.^93^ Such statements resonate with Gurib-Fakim’s claims about the apparently ‘uncontested’ nature of knowledge production in Mauritius, but it controversially obscures and dilutes the contributions of subaltern healers.

In addition, the valuing of medicinal plants in Mauritius has been portrayed as inherently dependent on their translation and co-optation within scientific procedures that lie elsewhere. Regulations are expected to be aligned to facilitate this translation. But the effect of this translation is also a dissociation and uprooting from the everyday knowledges that surround them. The knowledge on which these practices rest is reframed as lay knowledge just as the scientific knowledge to come is portrayed as expert, reproducing colonial logics that distinguished ‘know-how’ from ‘scientific knowledge.’^94^ All in all, if plants are to be seen as a different route into the recognition of a specific Mauritian tradition in healing, it is at the cost of human relations, overshadowing not only critical questions about the value of ‘creole’ Mauritian knowledge, but about the continuing devaluing of tacit knowledges that have not been translated by science.

Of course, the law is not always best suited to protecting the numerous healers who are side-lined from either policy or regulatory expectations. Instead, what becomes evident in this case is the rather the complicity of the law in filtering and re-ordering Mauritius’ healing landscape by creating new lines of legitimacy. Both the Ayurveda Act and the anticipatory discourses around medicinal plants were underpinned by parallel visions of a technical future for traditional medicine, of which some forms of categorisation and purification are a condition. In this way, both avenues partake in processes of decreolization: the legislation does this by purifying the meaning and scope of traditional medicine, while efforts to embed Mauritian plants within international scientific technical soft laws do this through the dispersion and reappropriation of creolised traditional medicines. Both legal imaginaries illuminate how the law is given the capacity to translate and purify the messiness of these social struggles. At the same time, these struggles to define the cultural and historical identity of traditional medicine in Mauritius also highlight the persistence of colonial logics, revealing the enduring vulnerability of creolised healing knowledges insofar as their ontological survival depends on their ability to adapt and transform.^95^ Holding in sight these different legalities is by no means a recipe for solving the complex ethical questions that arise in the governance of traditional medicine, but may create a space to question and explore their significance.

## Footnotes

1 Ethics approval granted by institution on the 1st of March 2017.

2 Cant (2020); Gale (2014).

3 Wahlberg (2014).

4 Ma (2010); Winterbottom (2021); Urquiza-Haas and Cloatre (2022).

5 Monnais (2021), p. 131.

6 Fassin and Fassin (1988).

7 Hokkanen and Kanonja (2019); Osseo-Asare (2014).

8 Brixius (2020); Gomez (2017); Schiebinger (2017).

9 Tilley (2021).

10 Ashworth and Cloatre (2022).

11 Hohmann (2016); Wahlberg (2014).

12 Soto Laveaga (2015).

13 Waddell (2020).

14 Wahlberg (2010); Pordié and Hardon (2015).

15 Zhan (2009).

16 Adams (2002).

17 Kloos (2017)

18 Blaike (2016).

19 Subramaniam (2019).

20 Subramaniam (2019); Kudlu and Nichter (2019).

21 Jasanoff and Kim (2009).

22 Langwick (2015), p. 493.

23 Harrington (2018), p. 236.

24 Harrington (2018); Foster (2014).

25 Monnais (2021).

26 Cloatre et al. (2023).

27 Cloatre and Enright (2017).

28 Silbey and Ewick (1998), p. 23.

29 Sussman (1981).

30 Indo-Mauritians represent 68 per cent of the population: 52 per cent are Hindu, and 16 per cent are Muslims. Creoles represent 27 per cent, and French/British only 2 per cent of the population. See: Kasenally (2011).

31 Sutton (2007).

32 Le Mauricien (2018) ‘“Rainbow Nation: Myth or Reality?’, https://www.lemauricien.com/le-mauricien/rainbow-nation-myth-or-reality/186274/.

33 Sutton (2007).

34 Laville (2000); Waters (2018); Boswell (2006).

35 Human Service Trust, Interview recorded in Calebasses, 4 December 2019.

36 Boodhoo (2015)

37 Rajkomar (2018).

38 In 2018, the Secretary of the Mauritius Tamil Temples Federation requested the inclusion of Siddha medicine to the Minister of Health and Quality of Life. See: Hansard, First Session, 10 April 2018. No. 03 of 2018, No. B/182, p. 177.

39 In 2002, the Ministry of Health and Quality of Life published a white paper announcing the introduction of Ayurvedic services to public health services. See: Ministry of Health and Quality of Life, ‘Health Sector Development and Reform’, https://health.govmu.org/Documents/Services/publications/whitepap.doc, 2002.

40 *Ayurveda and Other Traditional Medicines Act*, s 24.

41 Interview recorded in Mauritius on 5 December 2019.

42 High Commission of India, ‘Ayurveda Day’, https://hcimauritius.gov.in/statement?id=e73je, November 12, 2020; Prime Minister’s Office, ‘Cabinet Decision June 12, 2020’, https://pmo.govmu.org/CabinetDecision/2020/Cabinet_Decisions_taken_on_12_June_2020.pdf. At 8.

43 The Pharmacy Act 1983 and the Pharmaceutical Council Act of 2015 exempt these products.

44 Elaheebocus and Mahomoodally (2017).

45 A report by the Mauritius Competition Commission noted the absence of legal frameworks for the regulation of traditional medicines, as well as health supplements, cosmetics, and medical devices (2021). See: Competition Commission ‘Pharmaceutical Market Sector in Mauritius’, https://competitioncommission.mu/wp-content/uploads/2021/06/ms004-fullreport-080621, 8 June 2021.

46 Mahomoodally and Muthoorah (2014); Mahomoodally et al. (2019).

47 Sussman (1988); Sussman (1980); Luximon et al. (2019); Boaz (2020); Mahomoodally et al. (2016).

48 Hansard, Legislative Assembly of Mauritius, Public Bill, No XXXVI of 1989, para. 4139.

49 Colonial authorities in Mauritius passed in 1838 an act that banned fortune-tellers, diviners, and dream interpreters, followed by vagrancy laws in 1889 which prohibited people from pretending to practice witchcraft. These laws were adapted first for the Caribbean colonies, where their purpose was to stamp out indigenous religions, and in particular, slave witch doctors. See: Boaz (2020).

50 Cloatre et al (2021).

51 Easterby-Smith (2016); Brixius (2020).

52 Suroowan et al. (2019).

53 Pourchez (2017), p. 104.

54 Brixius (2020), p. 23.

55 Suroowan et al. (2019).

56 Eriksen (2007).

57 Craig and Gerke (2016), p. 92.

58 Vaughn (2005), p. 2.

59 Interview recorded in Mauritius on 4 December 2019.

60 Interview recorded in Mauritius on 22 November 2019.

61 Pordie (2007).

62 Masango (2020).

63 Hansard, Legislative Assembly of Mauritius, Public Bill, No XXXVI of 1989.

64 Srebrnik (2002).

65 Mukharji (2021), p. 95.

66 Mukharji (2021), p. 96.

67 Interviews in Mauritius recorded on 3 December, 2019; 22 November 2019; 4 December 2019.

68 Interview in Mauritius on 31 July 2018.

69 Interview in Mauritius on 6 December 2019.

70 Only 2 per cent of the island’s native forests have survived since colonisation. See: European Union External Action ‘Forests our lifeline’, http://eeas.europa.eu/eeas/forests-our-lifeline_nb, 31 March 2021.

71 Interview in Mauritius on 6 December 2019. See also: Boswell (2005).

72 Interview in Mauritius on 6 December, 2019.

73 Interview in Mauritius recorded on 3 December 2019. See also: Sussman (1980).

74 This project aimed to empower women to become leaders in biodiversity conservation through cultivating traditional medicinal plants. See, UNDP, ‘Grandma’s Secret: Empowering Women through Solar Powered Herbal Teas’, *GEF Small Grants Programme,* 2019, https://undp.shorthandstories.com/gef-sgp-grandmas-secret/index.html.

75 Gregory (1995), p. 475.

76 Suroowan et al (2019).

77 The remit of the Indian Ocean Commission, after its creation in the 1980s, had been largely the management and preservation of the environment and its biodiversity. The original participatory states included Mauritius, Madagascar, and the Seychelles.

78 Gurib-Fakim (1997); Gurib-Fakim (2019), p. 77.

79 The goal of the Centre for Phytotherapy Research was to become a company that would provide ‘innovative plant extracts and resources from a global biodiversity hotspot to the cosmetics, food, and pharmaceutical industries.’ See: Gurib-Fakim (2019), p. 109.

80 Association for African Medicinal Plants Standards, ‘Our History’, https://www.aamps.org/our-history.

81 As a European-African collaboration, the publication was sponsored by the European Union and the Centre for Development Enterprise in Brussels. See: Gurib-Fakim (2019), p. 183.

82 Clinical research on human subjects had been prohibited in Mauritius before the 2011 Act. See also: Gurib-Fakim (2019).

83 Mauritius is a member state of the Convention on Biological Diversity and the Nagoya Protocol on benefit sharing since 2012.

84 Gurib-Fakim (2017).

85 Hayden (2003), p. 119.

86 Pickersgill (2011).

87 Gurib-Fakim (2019).

88 Mukharji (2021).

89 Monnais and Tousignant (2016); Ashworth and Cloatre (2022).

90 Mawani (2009) p. 93.

91 Gurin-Fakim (2019), p. 110.

92 Eriksen (2007).

93 Eriksen (2007); Van der Werf et al. (2020), p. 6.

94 Murphy (2011).

95 Langwick (2011), p. 233.
